# Banned from the sharing economy: an agent-based model of a peer-to-peer marketplace for consumer goods and services

**DOI:** 10.1007/s00191-017-0548-y

**Published:** 2017-12-28

**Authors:** Adrien Querbes

**Affiliations:** Alliance Manchester Business School, University of Manchester, Denmark Road Building, Denmark Road, Manchester, M13 9NA UK

**Keywords:** User reviews, Decentralization, Complex adaptive system, Sharing economy, C63, Computational Techniques / Simulation Modeling; L22, Firm Organization and Market Structure; L86, Information and Internet Services / Computer Software; O33, Technological Change: Choices and Consequences / Diffusion Processes

## Abstract

The emergence of profit-based online platforms related to the Sharing Economy, such as BlaBlaCar and Airbnb, provides new means for end users to create an income from their possessions. With this opportunity, participants have to make strategic economic decisions despite limited formal expertise and information. Decentralization (using digital technologies) and reputation (using user reviews) are the core mechanisms chosen by these platforms to mitigate these limitations and to work efficiently as online matchmakers. We test the performance of these two mechanisms by studying the allocative efficiency (in terms of value and volume of transactions) of simulated marketplaces under different types of motivation from the participants and control from the platforms. As a result, we find an inverted-U relationship between the decision-making leeway available to the participants and the platform’s allocative efficiency. From the participants’ perspectives, too much freedom or too many barriers lead to market failures affecting specific participants: low-end consumers are banned from the marketplace while high-end providers experience lower levels of activity. As governance advice for these platforms, we show the limitations of promoting these platforms on the sole motive of monetary rewards.

## Introduction

The Internet has dramatically changed the production and distribution of consumer goods and services. Users have seized these new opportunities to bypass well-established businesses for production and trade. In this research, we focus on those online platforms that give anyone an opportunity to share their possessions for profit – such as airbnb.com, blablacar.com and turo.com. These platforms operate in a grey area between traditional marketplaces (supplied by formal firms) and the not-for-profit part of the Sharing Economy (Belk 2014). Consequently, specific forms of market governance have to be found in order to provide an efficient platform that would embed both profit motive and the willingness to share.

Due to its rapid growth, this sector has been largely scrutinized in the media and in the academic literature. Codagnone et al. (2016) provide an extensive literature review showing how the potential of these platforms is widely assumed to be correlated with regulatory matters. In the same line, Frenken (2017) sketches three potential futures of sharing based on different institutional decisions by the markets, governments and the public. Here, we take a different approach by showing how economic governance at the platform level alone can provide predictions of the potential successes and failures of the platforms. By doing so, we question the ability to provide economic governance by relying only on intermediation technologies. We show that the online platforms of the profit-based sharing economy are not neutral technologies. Only specific forms of governance over the transactions can help the platform to meet the expectations of the sharing economy to benefit all participants. The focus on internal governance supplements the widely discussed external factors of platform success and failure, e.g., the different levels of government regulation, the competition with other platforms or incumbents, and their social legitimacy.

Our interest in this issue comes from the emergence of a hybrid form of governance between intermediation and community building. On the one hand, these platforms define themselves as ‘intermediaries’, creating an apparent proximity with other online intermediaries such as eBay.com, Craigslist or Facebook, because of a strong technological integration with the Web 2.0 (including social networks and user reviews) (Belk 2014). The main difference between intermediaries and merchants is the distribution of control between the platform and the participants (Hagiu 2007). While the merchants keep full control over the sale (e.g. pricing, quantities), intermediaries only control the affiliation to their platform, taking the form of a two-sided platform connecting independent sellers and buyers. On the other hand, by putting forward the idea of sharing, these platforms position themselves in a moral system interested in social welfare (Botsman and Rogers 2011). Assuming that learning and enforcing morality is the result of both individual attributes and social relations (Hodgson 2014), decentralized platforms do not provide the structure for this kind of social enterprise. For instance, we can observe this ambiguity in the governance of Airbnb, in which the platform emphasizes its role of intermediary by describing itself as “a trusted community marketplace”^1^ rather than a company or a merchant. Yet, on September 8th 2016, Brian Chesky, CEO and co-founder of Airbnb addressed an email to the entire community of users entitled “Discrimination and Belonging”.^2^ The core of the email consists in the acknowledgment of discrimination on the platform, and the description of new platform policies implementing stronger control over the users’ behaviors.

In this paper, we study the interaction of platform governance and participants’ behavior using an agent-based simulation model. The model consists in the formal representation of a marketplace where we simulate pricing and purchasing decisions by the participants. Our objective is to study the specific conditions under which the online platforms of the sharing economy can approach the allocative efficiency of a clearing house (i.e. a fully-informed and fully-centralized intermediary that generates the maximum value and volume of transactions). As an intermediary, the platform governs the diffusion of information between the participants. This is the central component of the model that simulates it via mechanisms controlling what information is accessible to the participants so as to copy the search engines available for the buyers to find a seller. In addition, we assume that purchases and user reviews are the main sources of information available to the sellers. At the individual level, the model implements behaviors by assuming that actions are motivated by monetary or non-monetary incentives, also known as extrinsic and intrinsic motivations (Benkler 2004). The original work by Frey and Jegen (2001) studies the positive and negative influences of extrinsic motivation (monetary incentives or punishments) when the action was originally performed using intrinsic motivation. This paper aims to provide a better picture of the potential evolution of the platforms, in line with the recent concerns that emerged about the predatory or greedy nature of the for-profit platforms of the sharing economy (e.g. Frenken and Schor 2017; Slee 2016).

As a result, this modelling framework gives us the opportunity to study the interplay of specific structures of platform control and individual motivation. For each structure, the simulation shows patterns of allocative efficiency from two perspectives: the platform perspective (aggregated level) and the participant perspective (individual level). In a nutshell, the limited information generates local market frictions. The individual adaptation to these frictions leads to specific types of failures at the aggregated platform level, depending on the participants’ motivations. From the platform perspective, the market failure is more systematic when participants are only extrinsically motivated and when control is either too constraining or completely absent. From the participant perspective, we are able to specify which categories of participants and transactions are banned from the platform: the low-end of the demand side cannot access the marketplace due to the overpricing of the low-end of the supply side, and the high-end of the supply side is undervalued. The simulation results also give us the opportunity to discuss insights for platform policies to increase successful trading. This view of platform control and individual motivation is based on Landini (2016), who describes this evolution as a key characteristic of the evolution of the digital economy toward one equilibrium of high control and extrinsic motivation. However, our mechanisms of control are more limited: platform owners can never achieve full information about the participants. They cannot control the timing of purchases, they can only influence the information available for that decision.

## Literature review

In this paper, we limit our analysis to the sharing of consumer goods that Benkler (2004) describes as ‘shareable goods’. Those goods are ‘lumpy’, which is equivalent in economics to ‘indivisible’, to the extent that once acquired, the utility or functions provided by these goods cannot be changed gradually. In addition, they are ‘mid-grained’, meaning that “there will be relatively widespread private ownership of these goods and that these privately owned goods will systematically exhibit slack capacity relative to the demand of their owners” (Benkler 2004: 276). Accommodations and cars are examples of shareable goods that can be found on platforms such as airbnb.com (renting accommodation), blablacar.com (ridesharing) and turo.com (car sharing). On all these platforms, individuals can share their under-utilized possessions with other individuals in a peer-to-peer system. Other examples include household goods (such as bikes, power tools or ladders) that can be rented on neighborgoods.org or parking spaces on justpark.com.

According to Rachel Botsman,^3^ a platform belongs to the ‘sharing economy’ when: (1) the under-utilized assets are used more efficiently by users who specifically value access over ownership; and (2) the platform owner / designer and the participants exhibit values such as transparency, respect or belonging. Botsman does not limit the sharing economy to the profit-based platforms: under-utilized assets can be traded for monetary or non-monetary benefits. By focusing our own research on platforms using monetary transactions, we want to understand the behavioral interplay between monetary incentives (sharing for a price) and non-monetary incentives (e.g. sharing for the public good) that emerge on these platforms.

### The motivations to join and participate in the sharing economy

Shareable goods are probably as old as private property. Sharing practices also pre-date the Internet: homeowners renting spare rooms, cooperatives, etc. (Belk 2014; Frenken and Schor 2017). Yet, with the commoditization of the Internet, the extent of sharing entered a new phase of evolution. According to Benkler (2004: 278), “technology does not determine the level of sharing. But it does set threshold constraints on the effective domain of sharing as a modality of economic production. Within the domain of the feasible, the actual level of sharing practices will be culturally driven and cross-culturally diverse.” Technology has reduced the transaction costs of sharing, making peer-to-peer transactions more attractive.

From an economic perspective, the literature on two-sided platforms (e.g. Eisenmann et al. 2006; Rochet and Tirole 2006) provides a major contribution to the understanding of the economic issues faced by online platforms. Even if not limited to the Internet, it addresses the classic ‘chicken and egg problem’, i.e. the indirect network effect of two-sided platforms: to grow, the platform needs sellers to attract buyers, and vice versa (Caillaud and Jullien 2003). Firmly embedded in Industrial Organization, the strategic decisions are located at the platform level: the success of a nascent online platform relies on its ability to compete efficiently against other platforms or against brick-and-mortar incumbents. The solution to these issues is typically achieved by pricing structures, or rules and regulations. The pricing structure can take different forms. One side of the platform can be subsidized, while the loss is compensated by the other side, e.g. ‘divide-and-conquer’ (Caillaud and Jullien 2003). One can separate the price of access and the price of usage: e.g. by using fixed fees and variable fees (Rochet and Tirole 2006). The rules and regulations can be at the platform level, for instance, by regulating multi-homing (Armstrong 2006), or the bundling of complements (Eisenmann et al. 2006). The regulations can also be public, for instance, platforms can face antitrust concerns (Rysman 2009). However, as Levin (2013: 57) puts it, this is a ‘high-level view’ of Internet platforms, “focusing on platform pricing and user decisions to join a platform, but not addressing how platforms try to structure economic or social activity in ways that create value.” For instance, Bailey and Bakos (1997) find in the literature four reasons to use online intermediaries: aggregation, trust, facilitation and matching. Here, joining the platform would only be one aspect of aggregation.

Even if the rest of this literature review will be moving toward a more disaggregated view of online platforms, we must acknowledge the contribution of the two-sided platforms literature for our research question. By looking at the network effect and externality, this literature put forward how individual decisions (such as joining a platform) have consequences that we must include in the analysis of the platform strategy. In addition, even if pricing is only one instrument for the platforms to regulate individual behaviors and prevent market failures (Boudreau and Hagiu 2009), we still make pricing an important mechanism in our model. The major difference is that, on the peer-to-peer platforms of the sharing economy, pricing decisions are in the hands of participants and relate to individual transactions, not to the access and usage prices of the platform.

This change of focus corresponds to a different historical stage of the online platforms: while the two-sided market literature was interested in the attractiveness of nascent platforms (bringing more participants), we are interested in the sustainability of the platforms (keeping the participants satisfied). Hence, we supplement the analyses in terms of growth, competition and external regulation by looking at the framework surrounding the individual decisions once on the platform. This particular question does not benefit from the same level of empirical investigations and theoretical discussions, probably because of the focus on centralized platforms where individual decisions are mostly influenced by profit. A common denominator in this very small literature is to assess the behavior in terms of monetary versus non-monetary motivations. An early attempt by Benkler (2004) consists in identifying (1) the non-monetary motivations to perform specific actions in a sharing organization, and (2) the interplay between monetary and non-monetary motivations. The latter has been studied in behavioral economics by Frey and Jegen (2001) who report evidence of both positive and negative interplay of the two motivations, named intrinsic (non-monetary) and extrinsic (monetary).

Among the few studies collecting data on participants in profit-based platforms of the sharing economy, we note (1) a coexistence of intrinsic and extrinsic motivations, and (2) extrinsic motivation is what motivates the decision to join the platform, while intrinsic motivation explains the actions performed on the platforms. If the two types of motivation seem to reinforce each other positively, there are several views on their relative power. Bellotti et al. (2015) find that owner / designers of the platform are intrinsically motivated, unlike the participants who are extrinsically motivated. In the same line, for Codagnone et al. (2016), intrinsic motivation is mostly a characteristic of the early not-for-profit platforms. Lampinen and Cheshire (2016) observe that intrinsic and extrinsic motivations are adjusted by the participant depending on their current context, and even when the original motivation was not intrinsic, ancillary or unexpected intrinsic benefits are discovered once on the platform. Hamari et al. (2016) observe a predominance of intrinsic motivation, while Möhlmann (2015) finds first an extrinsic motivation (money saving) followed by trust (an intrinsic motivation). Böcker and Meelen (2017) provide a more precise picture of the diversity of motivations by accounting for different types of shared goods: quite intuitively, car-sharing is more environmentally-motivated, while sharing meals has a strong social motivation and sharing accommodations is economically motivated. Finally, Hellwig et al. (2015) identify four clusters of sharing consumers (idealists, opponents, pragmatists, normatives) whose motivations correspond to articulated beliefs rather than simple demographics. This is supported by Möhlmann’s observation (2015) that the decision to participate in a sharing experience involves rationalization and complex decision processes. This diversity encouraged us to model different scenarios regarding the relative hierarchy of motivations.

### The design of a decentralized matching market

By focusing on the individual participants rather than the platform, we need to adopt a performance measure that will reflect this perspective (instead of plaform market shares or winner-take-all competition). We do so by adopting the concept of “allocative efficiency” (Gode and Sunder 1993, 1997). In its most generic form, “allocative efficiency is the ratio of the actual to the potential gains from trade” (Gode and Sunder 1997: 603). The upper bound of the “potential gains” is reached when the participants can make all the transactions they desire, either as suppliers when they share their possessions, or as customers by accessing the shared goods. Concretely, on the demand side, Airbnb is functioning efficiently if the guests find a room when they desire to travel or BlaBlacar is efficient when there is a seat available for one’s desired journey. On the supply side, both the revenue and the efficient utilization of our shareable possessions are impacted if an Airbnb host or a BlaBlaCar traveler cannot find a buyer. From the public good perspective, such a failure can be seen as proof that profit-based platforms cannot provide the basic level of service required by Universal Service obligations (Slee 2016) or provided by incumbent firms (Cusumano 2015).

To achieve allocative efficiency, these marketplaces rely on decentralized governance using platform rules and complex peer-to-peer mechanisms designed to facilitate and control the transactions. However, both sides are affected by information scarcity, due not to the opportunism of the participants, but because of the difficulty to assess the exact content of the transaction (Benkler 2004). Usually, such uncertainty is reduced by encouraging the homogenization of products (norms, expectations of distribution channels or externalization of components) and the homogenization of tastes (marketing and conformity). In a peer-to-peer market, the variety of products and tastes might be overwhelming (Lu and Kandampully 2016), making it very difficult (1) for the buyers to assess the quality of each product, and (2) for the sellers to assess the size of the demand. If it is not possible to change the level of information or the degree of heterogeneity, versioning is the solution (Shapiro and Varian 1999): the pricing should reflect the heterogeneity by proposing different levels of price for different matches. Bhargava and Choudhary (2004) discuss how a menu of differentiated services (such as “freemium”) improves the allocative efficiency in a market characterized by heterogeneous users. However, if the versions are not clearly differentiated, efficiency diminishes due to the cannibalization of higher versions by lower versions. Marketing strategies can be used to magnify the product differentiation (for instance, by promoting exclusive or private access to specific categories of users), creating artificial search frictions. The interplay of search frictions and product differentiation is well understood in the industrial organization literature (Wolinsky 1986): product differentiation can cause imperfect competition if it creates an unequal distribution of market power among the providers and search frictions can exacerbate this by diminishing artificially the substitutability between products. As a consequence, search frictions provide a rational explanation of price dispersion (Chade et al. 2017; Kim et al. 2010; Levin 2013). However, the dispersion can reflect the improved allocative efficiency of versioning, as well as reflect uncompetitive pricing (Anderson and Renault 1999; Armstrong et al. 2009; Weber and Zheng 2007).

The objective of the literature on two-sided matching (Roth 1982) is to solve this matching problem in a more inclusive fashion. A matching problem arises when one attempts to match agents from two disjoints groups. Different algorithms or institutional arrangements can be used in order to perform the matching. This branch of research has contributed to the advance of solutions to the matching problem: theoretically (e.g. decentralized decision-making, information disclosure or bounded rationality) and with concrete applications (e.g. organ donors, assignment of children to schools). However, only a very limited amount of research has been conducted on situations where the available information and the preferences of the agents are still evolving (Chakraborty et al. 2010). In this study, the authors find that the interdependency between available information and preferences prevents stability in matching mechanisms. Such a situation is characteristic of emerging markets when product categories have to be invented and adopted by stakeholders, thanks to the experience gathered after repeated matchings (Suarez et al. 2015).

Using the case of labor economics, Anderson and Smith (2010) study a matching problem where the hidden characteristics of individuals are approximated via a reputation system. The reputation is updated by learning the individual characteristics through matchings. They discuss the necessary conditions and potential deviations of a perfect matching. A striking result is that, even if a stable matching can emerge, the matching mechanisms fail for specific levels of individual characteristics. However, they assume that reputation converge toward the real value of the characteristics. This limitation is inherent in some assumptions of the matching literature, such as the existence of a social planner and the use of delays (e.g. clearinghouses or deferred acceptance). Chade (2006) provides a solution to the absence of social planner or auctioneer by using two-player games, in a context of experience-based information and search frictions. He also finds a correlation between the player’s characteristics and its ability to find a match. This result is central to our own research: exclusion does not happen randomly, yet this author uses a marriage analogy limiting the reusability of information for repeated matching. (Post-marriage reviews are not available and the matched partners exit the market.)

The tradition of micro-founded market simulations (Gode and Sunder 1993; Kirman and Vriend 2001) provides an alternative set of solutions to this problem. This approach differs from the classic hypotheses of profit maximization, Walrasian auctioneer and perfect information, due to their lack of plausibility (Axtell 2005). Instead, heterogeneous agents learn, adapt and interact repeatedly, leading to the emergence of aggregate dynamics that cannot be entirely deduced from individual behaviors (Delle Gatti et al. 2012). Gode and Sunder (1993: 120) build on Vernon Smith’s work to establish that the “performance of an economy is the joint result of its institutional structure, market environment, and agent behavior. Institutional structure is defined by the rules that govern exchange, market environment by agents' tastes and endowments of information and resources, and agents' behavior by their trading strategy”. The implication is that the standard microeconomic assumptions (e.g. Walrasian auctioneer, profit maximization) can be implemented as a specific case within this framework.

A caveat is that this literature is mostly interested in testing different auction mechanisms (Marks 2006; Phelps et al. 2008) giving an extra emphasis on the negotiation phase, at the expense of the search phase. However, this stream of research provides results qualitatively similar to our research. Zhan and Friedman (2007) study a continuous double auction mechanisms where individual participants strategically adjust their profit motives (via markups and markdowns on their bids and asks) over repeated auctions. They find that market efficiency increases when there is a moderate markup by buyers and markdown by sellers and it decreases when it is more or less than moderate. Their explanation is that “in increasing the markup, traders face a tradeoff between greed (larger profit if there is a transaction) and fear (a greater probability of failing to transact)” (Zhan and Friedman 2007: 3000). Rud and Rabanal (2016) compare posted prices and decentralized (random) matching using the previous model. Posted prices do not achieve the same level of allocative efficiency as auctions. This result matters because posted prices are now the main form of pricing in the for-profit platforms of the sharing economy, auction systems being progressively replaced (Slee 2016) due to higher transaction costs (Levin 2013). Taking a more global perspective, Phelps et al. (2008) advise designing the market mechanisms very specifically taking into account the co-evolution of traders’ structure / characteristics and the matching problem.

Because both the matching market and the micro-founded literatures ignore search frictions, they have some limitations for our own research. The latter takes bounded-rationality and limited information as granted and, therefore, decisions are made using learning mechanisms. Yet these learning mechanisms are intended to improve the quality of the information used for the decision, in the same way that matching markets require information disclosure. In addition, both literatures assume the existence of a clearing-house, all transactions being processed synchronously. An alternative model of a “bazaar economy” with asynchronous transactions has been proposed by Miller and Tumminello (2015), establishing that search frictions are a secondary problem if one can control the distribution of traders’ characteristics. Such a possibility is not implemented in our model, but this form of control appears in the discussion.

### Trust and reputation

Kirman and Vriend (2001) – in their analysis of the dynamics and emergent properties of Marseille’s fish market – use the concept of loyalty to facilitate coordination and learning over repeated transactions. Loyalty is particularly relevant for the sharing economy, where social and non-anonymous relationships shape the transactions. However, with the Internet, the online platforms extend sharing to individuals outside our social circle and networks (Schor 2016). Most of the transactions involve new participants who are not going to enter a cycle of repeated transactions with the same partners and goods that cannot be examined before the transaction (Levin 2013). As Einav et al. (2016: 616) put it: “peer-to-peer businesses usually have to tradeoff between two important objectives: designing market mechanisms that efficiently elicit and incorporate dispersed information, and minimizing search and deliberation to keep the user experience convenient.”

The solution adopted by most platforms consists in asking the participants to write a post-transaction review. The aggregation of user reviews provides a basis for the creation and sharing of market information. This is a solution to some of the requirements of the matching problem (revealing preferences and hidden characteristics) using a simple mechanism. Also, the platforms depend heavily on user reviews because they help to build trust and reputation. Users are expected to give credit and contribute to reviews, mostly to determine the quality of goods that would be considered as ‘experience goods’ and, therefore, to oppose opportunism. Facing imperfect information about quality, the literature predicts various nuisances for customers: market concentration, limited incentives to supply the highest quality, overpricing. (See for instance Shapiro 1982 and Allen 1984.) Other solutions could address these nuisances. For instance, auctions are meant to punish greedy behaviors and misrepresentations of information to improve trustworthiness (Phelps et al. 2008). In the same line, an insurance system can be designed to prevent agency problems (e.g. moral hazard) when the content of the transaction can be strategically altered (Roger and Vasconcelos 2014; Weber 2014). However, these mechanisms expect the motivations to be purely monetary, i.e., the participants only fear a loss of profit. User reviews are more appropriate in the sharing economy because they also embed pieces of information that can be used for the non-monetary aspects of the transaction.

However, due to their simplicity, user reviews might not always prevent market frictions and they can sometimes aggravate them. In particular, user reviews produce similar concerns as product differentiation on the imperfect pricing of products (Armstrong et al. 2009). Reasons can be found in the literature review by Dellarocas (2003) who finds a rather general consensus that positive reviews lead to higher prices. At the same time, he shows how reviews have to be handled cautiously, because (i) a critical mass of reviews is necessary to derive a strategy, (ii) platforms control how the reviews are provided, and (iii) reviews lack context. The importance of the critical mass has been confirmed by Duan et al. (2008). They put forward “the dual nature of online user reviews”: (i) “consumer’s assessment of product quality”, and (ii) “product awareness among consumers”. They find that higher ratings do not affect the sales, while the number of reviews is correlated with sales (cf. bandwagon effect). For Hu et al. (2006), reviews are bimodal and give a true picture only when everyone answers. At the same time, user reviews reflect the opinion of a given person at a given time. This is the reason why, for Benkler (2004), user reviews incorporate the social aspect of sharing, but remain vague in terms of information processing. We assume that user reviews are central components in the moral system of the sharing economy, by providing the social structure for the “inculcation and enforcement” of values in the group together with individual preferences (Hodgson 2014).

In a nutshell, the profit-based online platforms of the sharing economy make the promise to build functioning marketplaces relying mostly on technology and values. The technologies behind the marketplaces use decentralization and user reviews as a form of control, information processing and gathering. In the rest of this work, we will analyze the conditions under which those technologies decrease or amplify the local frictions generated by the participants’ behavior.

## Model

Our model captures the operation of a peer-to-peer marketplace guided by decentralized decision-making and user reviews. Our objective is to compare the performance of different motivations when the peers try to complete transactions.

We present our model as an online platform for accommodations (such as Airbnb): the customers are ‘guests’ who want to find a ‘host’ for a night at a given location. Below, we describe the model in three blocks / sub-sections:

1. the governance of the marketplace;
2. the guests selecting an accommodation and writing a review;
3. the hosts adjusting their pricing decision.

**Fig. 1:**
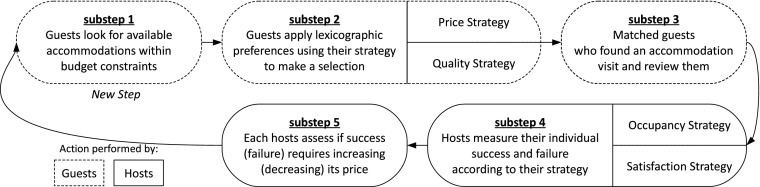
A typical time step of the simulation

Figure 1 gives an overview of the simulation by showing the five sequential substeps happening during every time step of a simulation.

### The marketplace

Gode and Sunder (1993) summarize some assumptions of micro-founded market simulations that we use in our model:

- demand and supply functions can be derived from simple budget constraints rather than maximization-behavior based on costs;
- the budget constraints being private, the demand and supply functions are unknown to the participants;
- the transactions occur on single units only.

By giving the opportunity to anyone of supplying consumer goods, peer-to-peer platforms encourage individuals to make a profit from their existing possessions. Even if the platform policies may advise them to mimic professional standards and norms, supplier costs are mostly opportunity costs related to the willingness to make transactions with strangers (Zervas et al. 2017). This is why we adopt the following assumptions about the marketplace:

- the *quantity* is capped (e.g. number of guests in an accommodation, number of seats in a car traveling a specific route);
- the *quality* is fixed (we assume that the possessions are not acquired solely for the purposes of being shared; they are under-utilized possessions);
- the *price* is related to personal motivations or constraints more than production costs;
- there is no *bargaining*; price adjustments are made sequentially, via experimentations made outside the transactions.

The marketplace comprises two categories of agents: a set of hosts (*H*) and a set of guests (*G*). Each agent is initialized with a fixed personal characteristic: for a guest, this is her budget (*b*), i.e. the limit of the monetary price she is willing (or able) to pay for the accommodation; for a host, this is the quality level of his accommodation (*q*) (this includes the characteristics of the host, too). Both *b* and *q* are distributed uniformly in [1100]. Our objective is to provide the platform participants with a personal and ordinal characteristic. Besides wealth and quality, this characteristic can be replaced to study the distributions of other critical characteristics, such as the distance from the city center or the willingness to pay for or to supply any service. By setting *b* and *q* with the same values, an omniscient matchmaker could centrally achieves a full matching where each guest could find a host with *p* = *q* = *b* (assortative matching where guests with budget *b* are matched with hosts of quality *b* = *q*). This full and centralized matching will be used as a benchmark to compare the allocative efficiency of the decentralized governance and differently motivated behaviors. We also assume that, at each simulation time step *t*, the exact same number of hosts and guests look for an accommodation: |*H*| = |*G*|. Consequently, all the hosts and guests are active at every time step, i.e., there is neither systemic shortage nor surplus on either side of the platform. This assumption is necessary for the platform to achieve full matching in the case of a centralized and assortative matchmaker.

For the guests, in our simple design, the fair price of an accommodation is *p* = *q* (i.e. 1 unit of price = 1 unit of quality). For individuals whose motivation is non-monetary, this simple assumption is also a social norm that is part of the non-monetary reward: visiting a host where *p* ≤ *q* will be seen at the individual guest level as a sign of respect and socially as a shared understanding of the social norms. This is the source of the interplay between monetary and non-monetary motivations in the model. The motivations of the participants are implemented via four scenarios described in Table 1. A given scenario connects a guest strategy (i.e. the type of motivation involved in choosing a host) with a host strategy (i.e. the type of motivation involved in adjusting the price). For tractability of the results, we assume that within a scenario all the hosts follow the same host strategy and all the guests follow the same guest strategy. Relaxing this assumption, for instance, by allowing a mix of strategies in a given period or over time, would require the comparison of a much larger number of scenarios.

**Table 1:** Scenarios based on host and guest strategies

		Guest strategy	
		Quality	Price
Host strategy	Satisfaction	Scenario I	Scenario II
	Occupancy	Scenario III	Scenario IV

When the simulation starts (at time step *t* = 1), the hosts do not know how to evaluate their accommodations. Hence, they randomly set a starting price *p*_1_ = (1 − *γ*) × *q* + *γ* ∗ *p*_*R*_ where ${p}_R\sim \mathcal{U}\left(\mathrm{1,100}\right)$ is a random component of the price, *q* is the fair price and *γ* balances the repartition between random and fully informed pricing. Then, at every simulation time step *t*, each guest will try to find an accommodation (substeps 1 and 2, see Fig. 1). If she succeeds, she will write a review regarding her experience of the accommodation quality (substep 3). Once the time steps are over, the hosts have the opportunity to adjust their price (substeps 4 and 5).

### Demand side: Booking and reviewing

The process of finding an accommodation takes the first two substeps described in Fig. 1, for each guest (more details follow):

- substep 1: guest *g* ∈ *G* reduces the set of hosts *H* to a subset *H*_*g*_, limited to the hosts who are not already booked and whose current price is within her budget constraints (i.e. a given price range).
- substep 2: if *H*_*g*_ ≠ ∅, i.e. one or more hosts match the criteria of substep 1, she selects one host based on its attributes. The selection process follows one of two strategies: Price Strategy or Quality Strategy.
  If *H*_*g*_ = ∅, no hosts match with this condition, she is considered unmatched and her search ends. The simulation returns to substep 1 with another guest.

In more detail, we learned from the literature on user reviews that reviews contribute to reputation building and are being used as a proxy rather than a fair source of information. For instance, Fradkin (2014) analyzed search data from Airbnb and shows that potential customers filter the listings based on objective variables (mostly the location and maximum price) before browsing the remaining listings and evaluating more subjective characteristics (including the number and text of reviews). Such a distinction can be found in the model of an online market with a self-selection bias of user reviews built by Li and Hitt (2008). In their model, they distinguish two classes of attributes: the ‘search attributes’, which can be inspected before the transaction, and the ‘experience attributes’, which are specific to each customer based on its experience of the product.

Our model follows a similar approach with substep 1 using objective information and substep 2 being more subjective. We have two objective search attributes available for the guests to make a decision during substep 1: *b*_*g*_ is the available budget of the guest *g*, and for each host *h* ∈ *H*, the guest can observe the price *p*_*h*, *t*_, i.e., the price requested to reserve the accommodation owned by *h* during night *t*. Using these attributes, during substep 1, all the guests sequentially try to find a host whose price is below their budget constraint and within a certain price range (*r*). The price range *r* gives us the opportunity to create some variations inside the scenarios, by looking at the effect of guests deviating more or less from their budget. For simplicity, *r* is the same for every guest inside a simulation. At the end of substep 1, for each guest *g*, *H*_*g*_ is the subset of hosts from hosts *H* who are not yet booked and whose prices are below the budget *b*_*g*_, but not lower than *b*_*g*_ − *r*:

$$ {H}_g=\left\{h\in H|{booked}_h=\mathrm{false},{b}_g-r\le {p}_{h,t}\le {b}_g\right\} $$

Then, the specificity of our platforms of interest is that quality is both a search attribute (the quality described by the host) and an experience attribute (the quality experienced by the guest and assessed in the user review). Based on the literature reviewed above, we make the following assumptions about the quality *q*_*h*_ of each host:

- the hosts are unable to assess their own quality because shared accommodations are heterogeneous, non-standard and include the characteristics of the host;
- the guests are able to observe the quality once they visit the accommodation;
- this observation leads to subjective individual reviews.

This is the reason why, during substep 2, we implement two selection strategies, related to the concept of “lexicographic preferences” and developed by Valente (2012). Both strategies are based on an algorithm of product selection including empirically-validated hypothesis on the behavior of boundedly rational and adaptive customers. We implement it in its simplest version: products are defined by two characteristics (quality and price). Customers have an order of preference for these characteristics, in line with their motivation (monetary and / or non-monetary). For both strategies, if *H*_*g*_ ≠ ∅ after substep 1, then substep 2 has two components (substep 2.1 and substep 2.2), one for each characteristic. During substep 2.1, each guest creates a subset $\dot{H_g}\subseteq {H}_g$ by selecting the hosts with the best performance in her *first* characteristic of interest. Then, during substep 2.2, if $\left|\dot{H_g}\right|>1$, each guest creates a subset $\ddot{H_g}\subseteq \dot{H_g}\subseteq {H}_g$ by selecting the best performance in her *second* characteristic of interest. If $\left|\ddot{H_g}\right|>1$, the host is chosen randomly from $\ddot{H_g}$.

The selection strategies are the concrete implementation of the behavioral decision making made by the agents using their specific form of motivation. The quality strategy reflects an agent whose motivations are mostly non-monetary: the quality *q* reflects the objective characteristics of the accommodation, but in the case of the sharing economy, the quality includes immaterial characteristics related to the hosts such as friendliness, shared values and so on. The price strategy is only monetary, in the sense that the guest value the savings provided by the platform.

Based on the information available about the accommodations’ characteristics, we implement two different strategies for the guests to select an accommodation:

- **Quality Strategy**: the only way for guests to approximate the quality of an accommodation is to use the price as a proxy of quality, provided that the host has priced his accommodation adequately:
  - substep 2.1: among the hosts in *H*_*g*_, guest *g* selects the host(s) with the highest reputation (number of positive reviews) within a margin of tolerance (*τ*_*reputation*_), hence creating the subset $\dot{H_g}=\left\{h\in {H}_g\right|\ {Pos}_{h,t}\ge \max \left({Pos}_{i,t}\right)\times \left(1-{\tau}_{reputation}\right),\kern0.5em \forall i\in {H}_g\Big\}$.
  - substep 2.2: among the hosts in $\dot{H_g}$, guest *g* selects the host(s) with the highest price (as a proxy of the highest quality) within a margin of tolerance (*τ*_*price*_), hence creating the subset $\ddot{H_g}=\left\{h\in \dot{H_g}\right|\ {p}_{h,t}\ge \max \left({p}_{j,t}\right)\times \left(1-{\tau}_{price}\right),\kern0.5em \forall j\in \dot{H_g}\Big\}$.
- **Price Strategy**: Zervas et al. (2017) point up how Airbnb has affected a specific segment of the hospitality industry, the lower-end hotels. Hotels offering business services do not suffer from the emergence of this platform. Guttentag (2015) observes that Airbnb is attractive to the low end of the market and covers a demand that is unattractive for the incumbents of the hospitality industry. Airbnb provides accommodations cheaper than hotels. Our understanding is that saving money is a strong motivation on these platforms:
  - substep 2.1: among the hosts in *H*_*g*_, guest *g* selects the cheapest accommodation(s) within a margin of tolerance (*τ*_*price*_), hence creating the subset $\dot{H_g}=\left\{h\in {H}_g\right|\ {p}_{h,t}\le \min \left({p}_{i,t}\right)\times \left(1+{\tau}_{price}\right),\kern0.5em \forall i\in {H}_g\Big\}$.
  - substep 2.2: among the hosts in $\dot{H_g}$, guest *g* selects the host(s) with the highest reputation (number of positive reviews) within a margin of tolerance (*τ*_*reputation*_), hence creating the subset $\ddot{H_g}=\left\{h\in \dot{H_g}\right|\ {Pos}_{h,t}\ge \max \left({Pos}_{j,t}\right)\times \left(1-{\tau}_{reputation}\right),\kern0.5em \forall j\in \dot{H_g}\Big\}$ where *Pos*_*h*,*t*_ is the number of positive reviews for host *h* at the current simulation time step *t*.

Both strategies address the user reviews in the same way: they count the number of positive review as a way to distinguish between reputable and less reputable hosts (substep 2.2 of price-oriented strategy and substep 2.1 of quality-oriented). The differences are: the reversed lexicographic preferences between price and reputation, and the quality-oriented guests use the price as a proxy of quality while the price-oriented guests are focused on saving money. The tolerances *τ*_*price*_ and *τ*_*reputation*_ give us an opportunity to create some variations inside the scenarios, in the same way as *r* (the price range) does in substep 1, as did Valente (2012).

Once all the guests have had an opportunity to find an accommodation, the guests who have found a match move to substep 3. During substep 3, the strategies make no difference and no additional parameter is used. Each matched guest visits her host and she reviews the quality of the accommodation. The content of the review depends on the similarity between the price paid and the quality observed while staying. The platform stores the review as a binary grade.

$$ {M}_{h,t}=\left\{\begin{array}{ccccccc}0& \mathrm{if}& \mathrm{quality}& {q}_h& <& \mathrm{price}& {p}_{h,t}\\ {}1& \mathrm{if}& \mathrm{quality}& {q}_h& \ge & \mathrm{price}& {p}_{h,t}\end{array}\right. $$

In other words, 0 means that the guest was ‘unsatisfied’ by the value for money of the accommodation, while 1 means ‘satisfied’ or ‘positive review’. We use these simple grades in order to account for the difficulty to transfer valuable information from one guest to another (e.g. specific preferences, limited trust in strangers). Yet, following Hu et al. (2006), a bimodal system of user reviews is still efficient when every participant contributes a review. So the potential frictions initiated by user reviews are not the result of their incompleteness, but come from the manner in which they are used by the supply side.

### Supply side: Sharing profitably

Once substeps 1 to 3 are performed by the guests, the hosts have the opportunity to adjust their price. At substep 4, the hosts assess their individual successes and failures according to their own strategies. Reviews and occupancy form the only source of information they have to evaluate their accommodations. However, the guests had surveyed the market for their own selection process and, therefore, the selection outcome informs the hosts about their own market position. Depending on their strategy, they will define a success (*m*_*s*,*h*,*t*_) and a failure (*m*_*f*,*h*,*t*_) differently:

- **Satisfaction Strategy**: receiving a bad grade (*M*_*h*, *t*_ = 0) is a failure equivalent to not hosting a guest at all.
- **Occupancy Strategy**: the content of reviews have no influences on the host’s decisions, the only thing that matters is the number of hosted guests.

Table 2 shows how successes (*m*_*s*,*h*,*t*_) and failures (*m*_*f*,*h*,*t*_) are computed. On these peer to peer platforms, we assume that the motivations of hosts and guests follow very similar logics. Consequently, hosts who are only motivated by monetary rewards will focus their decision strategy only on ‘occupancy’, while hosts who also value the social dimension of the platform will take into account the more qualitative ‘guest satisfaction’ in their strategy.

**Table 2:** Computation of m_s,h,t_ and m_f,h,t_, using the information accumulated since the last price adjustment

		Failure	Success
Host Strategy	Satisfaction	m_f,h,t_= number of steps without positive reviews	m_s,h,t_= number of positive reviews
	Occupancy	m_f,h,t_= number of steps without guests	m_s,h,t_= number of guests

At substep 5, the hosts make a final decision about price adjustment. They compute the probability to adjust the price

$$ Prob{\left(\mathrm{adjust}\  \mathrm{price}\right)}_{h,t}=\frac{\left|{\mathrm{m}}_{\mathrm{s},\mathrm{h},\mathrm{t}}-{\mathrm{m}}_{\mathrm{f},\mathrm{h},\mathrm{t}}\right|}{a_{h,t}}\times \varepsilon $$

where *a*_*h*, *t*_ ∈ *ℤ* ^+^ is the number of steps since the last price adjustment (by definition, m_s,h,t_ + m_f,h,t_ = *a*_*h*,*t*_, i.e. successes and failures are measured since the latest price adjustment only). If m_s,h,t_ = m_f,h,t_, $\frac{\left|{\mathrm{m}}_{\mathrm{s},\mathrm{h},\mathrm{t}}-{\mathrm{m}}_{\mathrm{f},\mathrm{h},\mathrm{t}}\right|}{a_{h,t}}=0$, this means that, if successes equal failures, the host receives a mixed signal and doesn’t take action. If m_s,h,t_ = *a*_*h*,*t*_ or m_f,h,t_ = *a*_*h*,*t*_, $\frac{\left|{\mathrm{m}}_{\mathrm{s},\mathrm{h},\mathrm{t}}-{\mathrm{m}}_{\mathrm{f},\mathrm{h},\mathrm{t}}\right|}{a_{h,t}}=1$, this means that the host has the clearest signal possible that maximizes the probability of adjusting price. The rationale is that agents make decisions using all the (limited) information they receive from the environment. The probability captures the strength of the signal they receive: the more evidence points in the same direction, the more chance to follow this lead. This follows the minimalist modelling of cognitive reasoning used by Epstein’s (2014) agent_zero. Following Epstein, such a minimalist approach is still more realistic than giving the agents the ability to estimate the right price using a Bayesian approach, for instance. Consequently, the hosts try to estimate the probability of two events (overpricing, underpricing) using only the information accumulated since their latest action. Any older memory would be counterproductive in this minimalist setting since the information relates to a decision that has been changed.

*ε*, 0 ≤ *ε* ≤ 1, is a parameter to control the frequency of actions in the simulation. Its function is to prevent hasty decisions by forcing the hosts to delay actions. A random value is drawn from the uniform distribution between 0 and 1; if *Prob*(adjust price)_*h*, *t*_ is above this random value, the price is adjusted:

- if *m*_*s*,*h*,*t*_ − *m*_*s*,*h*,*t*_ > 0 (i.e. successes > failures), the price increases of 1 unit,
- if *m*_*s*,*h*,*t*_ − *m*_*s*,*h*,*t*_ < 0, (i.e. failures > successes), the price decreases of 1 unit,
- and, reminder, if *m*_*s*,*h*,*t*_ = *m*_*f*,*h*,*t*_ (i.e. successes = failures), no adjustment is made.

## Results

The results discussed below are based on the following parameters: population *P* = 200, *ε* = 0.2, host population *H* = 100, guest population *G* = 100. The results are recorded at *t* = 2000 time steps and they show the average over 40 repetitions of the simulation to control for the stochastic processes (i.e., host’s initial price, order of guest’s queue, host’s tiebreakers and host’s price adjustment).^4^ The degree of randomness of the initial price *γ* is 0.5 for brevity of the results and following the observation of its effects detailed in the Appendix. Inside the four scenarios (see Table 1 above), the price range (*r* = {1, 2, 5,10,15,50} for the aggregated data and *r* = {1, 5, 15, 50} for the individual data) and the tolerances (*τ*_*price*_ = {0.05,0.25} and *τ*_*reputation*_ = {0.05,0.25}) provide additional insights about the potential successes and failures of the platforms for |*r*| × |*τ*_*price*_| × |*τ*_*reputation*_| = 6 × 2 × 2 = 24 combinations of parameter values.

### Centralization versus decentralization of platform governance

We begin by analyzing the performance at the platform level. Taking the host perspective, we consider the aggregated *value* of all the transactions completed during the simulation, i.e., how much revenue was created for the hosts from their under-utilized possessions. Taking the guest perspective, we consider the aggregated *volume*, i.e., how many accommodations were booked and visited by the guests.

**Fig. 2:**
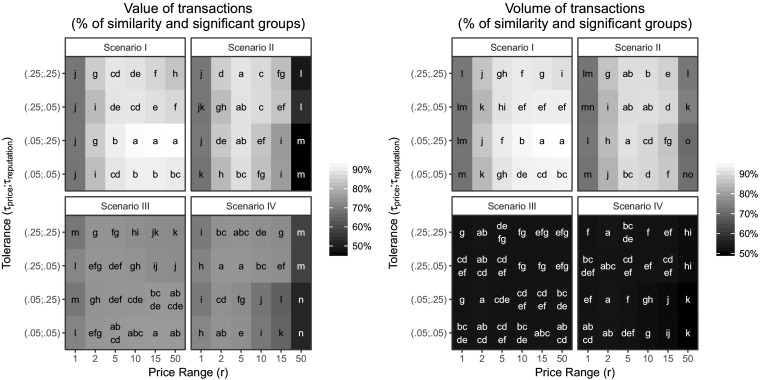
Aggregated value and volume of transactions at the platform level. For all the combinations of scenarios and parameter values, the figure compares the allocative efficiency relative to the benchmark (i.e, the centralized assortative matching) in terms of “similarity” with the benchmark. The efficiency is analyzed at the aggregated level for all the hosts in terms of “Value of transactions” and for all the guests in terms of “Volume of transactions”. The efficiency is analyzed using the data from the simulation (“% of similarity”) and a post-hoc analysis of this data (“Significant groups”). ”% of similarity” (graduated color scale from black to white): Percentage of similarity with the benchmark. For instance, a Value of transactions of 90% means that this specific configuration of scenarios and parameters has achieved to accumulate 90% of the value that would have been achieved by centralized assortative matching. “Significant groups” (letters “a” to “o”): For each scenario, percentages of similarity are compared in terms of statistical difference (ANOVA). Because they are significantly different, a post-hoc analysis is conducted to rank and group the similarities, using Tukey’s comparison of means at 99% confidence level. Two consecutive groups in the alphabetical order are significantly different at level 99%. For instance, “a” achieves a statistically significantly higher percentage of similarity than “b”. When the figure shows a string of more than one group in a single cell (e.g. “ab” or “cdef”), it means that the configuration of parameters of this cell does not produces a percentage of similarity significantly different from other cells in the same group(s)

Figure 2 shows the performances of the different scenarios, using the 24 possible combinations of parameters *r* (price range delimiting the budget constraint) and *τ*_*price*_ and *τ*_*reputation*_ (tolerances). The value and volume of the transactions are compared to the centralized assortative matching, where *p* = *q* = *b*. Such a benchmark has two meanings: (i) there is a perfect matching between hosts and guests providing a full and constant occupancy of the accommodations, maximizing the aggregated volume and value of the transactions (perfect allocative efficiency); (ii) the hosts have been able to adjust their price in order to reflect the actual quality of the accommodation. As a reminder, this requires a fully centralized market, where a fully informed matchmaker would assortatively match the guest with a budget *b* to the host with a quality *q* = *b*, as well as command the host to charge a price *p* = *q*. This is theoretically reachable in this decentralized model, because there is no shortage or surplus on either side of the market: *H* = *G*, as well as *q* and *b* have the same values among the hosts and guests, respectively. The challenge is for the hosts to discover the right price *p* of their accommodation via step-by-step incremental adjustments.

We make the assumption that the main objective of platform governance is to lead the participants closer to this benchmark. Figure 2 shows that Scenario I is the most successful means to reach this goal. In this scenario, hosts and guests are both motivated by the non-monetary signal embedded in the user reviews and they reach the highest levels of transactions in value and volume. Hosts receive 92–93% of the revenue and guests rent 95–96% of the accommodations they would have rented in a fully centralized system. In addition to the non-monetary motivation, the other condition is a wide price range and a greater tolerance for lower reputation (i.e. *r* = {10,15,50}, *τ*_*price*_ = 0.05 and *τ*_*reputation*_ = 0.25 identified in Scenario I by the significant group “a”).

The interplay of different forms of motivations, implemented via the hosts’ and guests’ strategies, is not one-sided. The level of performance of a given strategy is highly interdependent with the strategy adopted on the other side of the market. For instance, in terms of value, the Satisfaction Strategy by the hosts (Scenarios I and II) seems to be the most successful Strategy. However, a decision by the guest to adopt a Price Strategy (Scenario II), together with a wide price range, can lead the hosts to endure the lowest level of revenue (45–49%), while the guests still find 71–74% of accommodations. In terms of volume, the Occupancy Strategy by the hosts (Scenarios III and IV) bans nearly 50% of the guests from finding an accommodation. The individual search attributes (tolerance and price range) cannot influence this result. Yet, this strategy is not too detrimental for the hosts who receive up to 75% of the available revenue.

The price range *r* is the second most important factor explaining the performance of the platform.^5^ When *r* = 1, the guests potentially reveal they budget *b* by avoiding accommodations below their budget. In the two-sided matching literature, revealing this kind of information is essential for successful matchmakings. For this reason, platform owners might be willing to control the accommodation search in this way to mimic an omniscient matchmaker. However, Fig. 2 shows the poor result of this solution. In fact, we observe in many places an inverted-U relationship between *r* and performance (both value and volume of transactions). In most cases, both in value and in volume, the best performance is obtained for intermediate values (*r* = {2, 5, 10}). While *r* = 1 is clearly outperformed by *r* = 50 in Scenarios I and III, the reverse is true for Scenarios II and IV. The signal is clear for the platform governance: when the motivation is mostly monetary, it is better to limit the price scope during the guest search for accommodation, for instance, by putting the most expensive accommodations higher in the search results. Interestingly, the best performance described earlier includes *r* = 50. Consequently, there is a very fine line between the instrumentation of successful control and failed control.

Finally, the tolerances (*τ*_*price*_ and *τ*_*reputation*_) provide small but significant insights. The effect of *τ*_*price*_ is correlated with the guest motivation. When the motivation is not just monetary (Scenario I), increasing it reduces both the value and volume of transactions. On the contrary, when the motivation is purely monetary, a greater tolerance improve both the value and volume in Scenario II and the value in Scenario IV. The effect of a greater *τ*_*reputation*_ is positive when hosts are not just motivated by their occupancy. However, the benefits of tolerating a wider range of reputation decreases with the price range *r*. Consequently, a certain leeway is important on their first source of motivation observed on the platform, wherever the motivation is located in terms of side.

### Frictions among individuals on each side of the marketplace

**Fig. 3:**
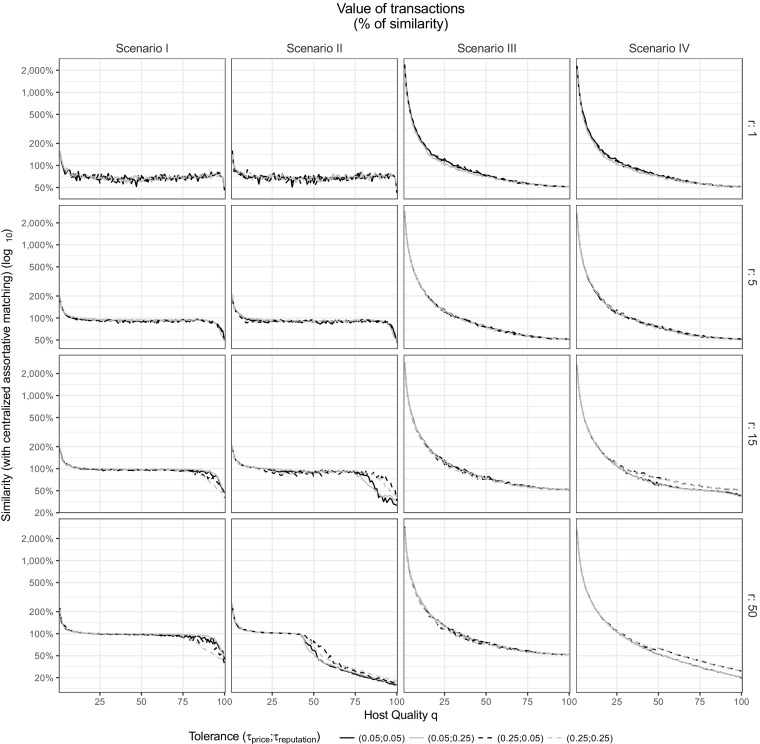
Value of transactions at the individual level for each Scenario. For each scenario, the graphs depict how much the marketplaces deviate from the centralized assortative matching in terms of value, depending on the tolerance and on the price range (***r*** = {**1**, **5**, **15**, **50**}, stacked vertically). Hosts are ordered by their unique and fixed quality ***q***

**Fig. 4:**
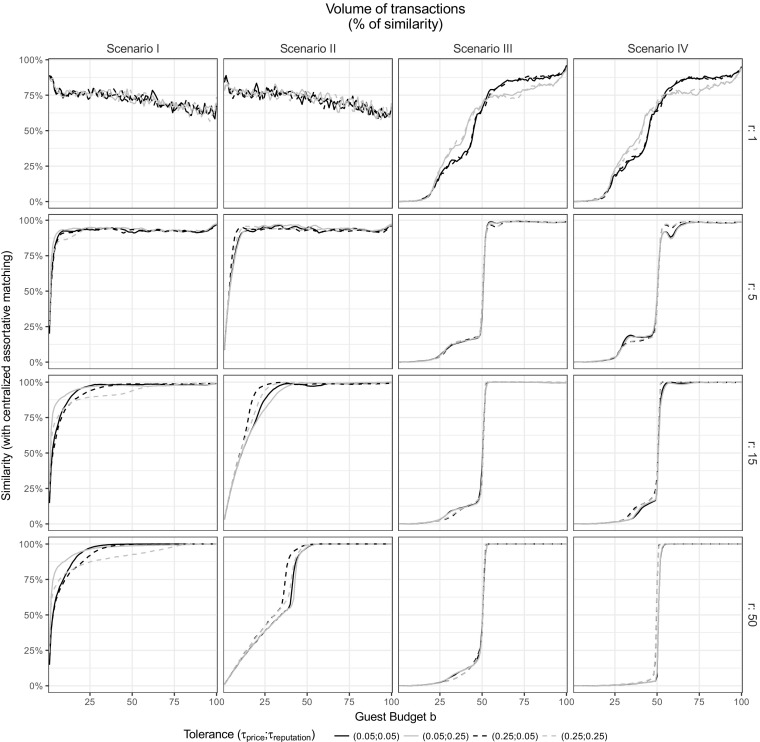
Volume of transactions at the individual level for each Scenario. Same as Fig. 3, in terms of volume. Guests are ordered by their unique and fixed budget ***b***

Additional insights about the performance of platforms are to be found in the frictions between participants inside the platform. In Figs. 3 and 4, the performance of the platforms (value and volume) is broken down by individual participants. For each host, ordered by quality level *q*, we individualize how much revenue (sum of values of transactions) received during the simulation, in percent of the revenue of full occupancy at *p* = *q* (i.e. centralized assortative matching, when the host accommodates a guest paying *p* = *q* = *b* at every time step). For each guest, ordered by budget level *b*, we individualize how many accommodations they are able to book, in percent of the full occupancy (i.e. centralized assortative matching, when the guests find an accommodation at every time step).

For every scenario, the simulation produces two trends: when the host quality increases, his ability to earn an adequate income decreases (Fig. 3) and the guest’s ability to book all the needed accommodations decreases with her budget (Fig. 4). While these results relate to the simple behavioral assumptions of the model, the range of strategies and parameter values offers us several options to assist the platform and avoid such extreme outcomes.

First, on our platforms of interest, matches are exclusive during one time step: e.g., accommodations are booked for a given date and they become unavailable for concurrent use. This process creates local shortages on specific segments of the marketplace, particularly when hosts are still assessing the quality or price of their accommodations. These shortages can be seen in our simulation by the allocative inefficiency in Figs. 3 and 4. In details, we observe that, when the price range *r* increases, the value and volume improve for some participants (hosts with intermediate quality in Fig. 3 and guests with high budget in Fig. 4). In fact, increasing the price range reduces the local shortages and, therefore, it increases the number of transactions, since guests are less likely to be without a host after substep 1 (i.e. when they look for a host within a range *r* of their budget). While the volume of transactions in Fig. 4 cannot exceed 100% (i.e. maximum potential allocative efficiency, when each guest achieve exactly one transaction at every time step), a value of transaction above 100% in Fig. 3 means that hosts can overcharge their accommodations leading to a revenue exceeding what would happen by assortative matching.

Second, increasing the price range of search has a counterpart: the additional pressure on the cheapest accommodation and the devaluation of the most expensive ones. As observed at the aggregated level, regarding the value of transactions (Fig. 3), these phenomena are particularly visible for Scenarios I, II and IV. At the individual level, when the price range *r* increases, we observe an increasing value for the hosts with the lowest quality levels and decreasing revenue for the hosts with the highest quality. The value being a direct result of pricing, these results also means that the lowest quality hosts overprice their accommodations (*p* > *q*) while the highest quality hosts underprice their accommodations (*p* < *q*). The tolerances from the guests can slightly alter these phenomena as noticed at the aggregated level. We can see now that the effect of *τ*_*price*_ is limited to the intermediate and high ends of the supply side.

Third, these phenomena of overpricing and underpricing also have a direct effect on the volume of transactions (Fig. 4), leading to the exclusion of the guests with the lowest budget. The exclusion increases with the price range *r* and is more limited for Scenario I and II than for Scenario III and IV where we observe a clear cutoff around the guest budget *b* = 50. The tolerance has an interesting effect in Scenario III and IV that was invisible at the aggregated level: a higher tolerance for reputation slightly smooths the inequality of successful matching between lower and higher budgets.

## Discussion

With the simulations, we have shown how the local frictions emerging on decentralized platforms create specific patterns of aggregated market failure that we have measured in terms of allocative inefficiency (equivalent to underactivity and exclusion). In fact, agent-based simulations help us to understand the conditions for “non-transactions”, which are more difficult to capture empirically than the transactions. Transactions can only measure what is working, when the traders benefit from the transaction. However, to assess the potential evolution of the platform, it matters to understand the platforms’ failures, too. Following the three components of our theoretical framework, we will now discuss how the extent and the shape of market failures are influenced by (i) the interplay of forms of motivation (the scenarios), (ii) the instruments of governance (stylized by the tolerances and price range during search), and (iii) the imperfect information created by the user reviews.

### The motivations of sharing

If one assumes that the profit-based online platforms of the sharing economy provides an opportunity to make a profit and / or contribute to the public good by sharing idle possessions, one can also assume that achieving just one transaction improves the social welfare (Botsman and Rogers 2011). Looking at the patterns of exclusion for specific participants that we just described, the threshold between success and failure is to be discussed. A certain level of underactivity is intrinsically linked to our assumptions: absence of negotiation (search is directed using posted prices) and absence of stock (only one unit is available per trader). In this context, Chade et al. (2017: 34) establish that the solution can only by a trade-off where “sellers that post lower prices will attract more potential buyers and will therefore sell with a higher probability. Buyers who pursue lower-priced goods must accept lower trade probabilities since there are more competing buyers. With two-sided heterogeneity and complementarity, this trade-off plays an important role in the determination of the equilibrium sorting patterns.” Clearly, the motivations of the participants influences the direction of the trade-off between economic profitably (value) and social activity (volume).

Underactivity may be acceptable, because the platforms exhibit the characteristics of a ‘long tail’, as observed in e-commerce (Einav et al. 2016). This can also be seen in our simulation, via the strong inequality in revenue and activity among members. Participants may react to this inequality via two behavioral patterns, as identified by Ikkala & Lampinen (2014: 175): increasing the price as the number of positive reviews grows larger to filter the demand or, on the contrary, underpricing the listing to increase its visibility. By capturing these behaviors in our model, we show how the unequal demand cannot be reduced to a long tail phenomenon, since it has consequences on the price structure of the marketplace leading to the exclusion of categories of users. The long tail can be seen as an opportunity for participants who do not value visibility because they cater to the needs of specific participants. However, the other side of the coin is that the visibility can be captured by the “superstar effect”, where successful suppliers benefit from the reinforcement learning until price and quality become disconnected (Levin 2013). This effect can be exacerbated by user reviews, as we will discuss below.

Conversely, underactivity can be detrimental to the platform, either by increasing the complexity of search due to the profusion of unverified sellers or by generating a negative word-of-mouth about the platform. The hypothesis that participants keep using the platform even if it is not efficient is at odds with other models, such as Einav et al. (2016). However, by assuming that the sharing economy provides goods to individuals who cannot afford transactions on traditional marketplaces, we simulate the emergence of an overpriced low-end market for captive users. By offering more consumption opportunities to individuals who were excluded from the traditional marketplaces, the profit-based sharing economy may produce its own failures by excluding or overcharging its participants. These failures are contradictory with some of the non-monetary expectations of the sharing economy, such as improving social capital and trust, or helping less advantaged groups (Codagnone et al. 2016). In addition, due to the winner-takes-all nature of two-sided platforms (Caillaud and Jullien 2003), these failures can have dreary consequences once a platform monopolizes the market (Frenken and Schor 2017). By identifying the specific locations of failure, our simulations show why buyers on the lower end and sellers on the higher end should join alternative platforms or encourage forms of governance that would be less detrimental to them.

### The instruments of platform governance

The main challenge established at the individual motivation level is that the trade-off between volume and value affect different sides of the platform, unlike Zhan and Friedman’s model (Zhan and Friedman 2007) where the auctions help to balance greed (increase the profitability of the transaction) and fear (limit the chance to achieve a transaction). This result put forward the complex design decisions made at the platform level. For instance, with the occupancy strategy in Scenarios III and IV, sellers who are motivated by monetary rewards make decisions that are detrimental to their competitors, customers and the platform as a whole. Concretely, this form of motivation from the sellers excludes a large part of the potential buyers (only 50% to 53% of allocative efficiency in volume), yet they manage to receive up to 75% of the value. This means high revenue per transaction. If we assume that the sharing activities are a side-activity for the participants, it seems fair that the participants want to limit their commitment to the most profitable transactions. However, this strategy fails to provide a Universal Service (Slee 2016) and it can be easily taken over by incumbents who propose a wider scope of products (Cusumano 2015). The platform owners can also share those concerns. If they receive a percentage of the transaction value and they are willing to reduce the costs related to an increased activity on the platform, they might favor Scenarios III or IV.

By contrast, owners interested in attaining a critical mass of participants to compete with the incumbents or achieve Universal Service would like to recreate the conditions of Scenario I. As exemplified by Brian Chesky email, platform policies can be implemented to encourage the adoption of non-monetary motivations. Put in terms of motivation, platform owners can use strategically their market environment to crowd out or crowd in participation on their platforms (Frey and Jegen 2001). Using the literature on two-sided matching, obtaining information about the motivations of users can be useful ex ante to create the behavioral conditions represented by the scenarios. In the same line, information about the user characteristics (such as their budget and quality in our model) can also give an opportunity to the platform owner to limit the entry of participants who will systematically be excluded. By studying a few simple governance instruments in our model (tolerance and price range), we have shown that these instruments have non-linear and strongly interdependent effects. Designing these instruments of governance is difficult, which can explain the numerous changes and experiments performed by the platforms, as well as the misalignment between the motivations of the designers and those of the participants, as observed by Bellotti et al. (2015).

From the participant perspective, our results echo the work of Lauermann et al. (2017) showing that decentralized mechanisms can achieve allocation efficiency even when the participants ignore the exact market condition (including their own characteristics), provided that the search frictions are small. To diminish the search friction, the platform should use all the governance mechanisms (monetary, non-monetary and informational) at its disposal (Boudreau and Hagiu 2009; Phelps et al. 2008). For instance, Gode and Sunder (1997) face a similar problem when their double auction system cannot prevent the allocative inefficiency resulting from the displacement of intramarginal traders by extramarginal traders (who do not value the goods as much as the intramarginal traders). As a solution, they sketch an alternative market governance that almost mimics the mechanism of centralized assortative matching (e.g., bids and asks closer to actual constraints, delayed decisions queuing in a clearing house). In the same line, Anderson and Renault (1999) use search costs to explain the emergence of “tourist traps”, i.e., options with similar characteristics and largely overpriced, because “buyers do not shop around”. Different mechanisms can be tested to achieve such results. In addition to the non-monetary instruments proposed above and following the lessons from the two-sided market literature, the platform can regulate the behaviors monetarily by subsidizing the underactive participants using fees collected from their overpriced counterpart. The platform can also modify how the information is displayed, for instance, to alter the tolerance and the price range of the participants. In fact, Airbnb uses such a mechanism in the search ranking of accommodations.^6^

### User reviews and reputation

In this research, we have made the assumption that user reviews are a major piece of information for participant decisions on the platforms. By implementing user reviews in only three of the four scenarios (Scenario IV is extrinsically-motivated on both sides), we were interested in testing the complex interactions of user reviews and decentralization. The main difference between Scenarios III and IV is the use (or not) of user reviews by the users. We observe only a very small difference: with user reviews, the high-end hosts are slightly more rewarded. Yet the user review alone is unable to prevent unsatisfactory transactions. On the other side, with Scenarios I and II, the user review is a very efficient tool to help the hosts in assessing the quality of their accommodations. Even if our results are slightly more positive toward the user reviews (since every guest has to write one), it also confirms that user reviews have a limited value without context and quantity (Benkler 2004; Dellarocas 2003), the reason being that user reviews can exacerbate the artificial prominence of some participants in a complex way. In line with our results, we expect search frictions to create captive users, leading the prominent supplier to increase its price, while the competitors have to reduce theirs (Armstrong et al. 2009). If we try to diminish the search frictions, the situation can deteriorate because the improved substitutability between products might make the participants even more dependent on user reviews for their decision and therefore reinforce the market power of the prominent suppliers (Kim et al. 2010).

Finally, in our model, user reviews are written regarding an identifiable and fixed quality of the accommodation. Yet, on other platforms, the quality of the transactions can be based on more subjective characteristics. For instance, with carpooling, the characteristics of the car and seats have a minor importance for the transaction, by comparison to the driver’s behavior. We assumed earlier that user reviews are a way to enforce and inculcate the values, moral system and expectations of the social group; consequently a feedback can exist between the user review and the behavior being reviewed. In an extreme form of control, the platform can use the reviews to select the best participants. The implementation of this strategy would again depend on the position of the platform and its participants regarding the trade-off between higher volume of transactions versus higher value (or quality) of transactions.

## Limitations and possible extensions

Using an agent-based model mimicking the emergence of a profit-based platform for sharing consumer goods, we show how much these platforms must balance motivations, as well as control. Even if the individual simulations involve agents with homogeneous strategies, they give some clear insights about the platform governance, particularly to the platform owners interested in avoiding such extreme outcomes. In addition to the insights developed above, this research also contributes to an assessment of a theoretical framework that can be used to monitor the real evolution of these platforms and, therefore, contributes to the empirical understanding of the profit-based sharing economy. For instance, the collection of data should not be limited to the successful transactions: failed transactions involve specific profiles of participant that reduce the universality of these platforms.

In this current version, the simulations are performed on a closed platform with a population of agents having immutable characteristics. A dynamic version of the model, introducing the entry of new users depending on platform success and the exit of unsatisfied agents, would be a particularly interesting extension of the model. More than an extension, adding dynamics in the population of participants relate to a different set of empirical and theoretical questions. The type of motivations / actions discussed here would have to be replaced by an analysis of the mechanisms triggering actions. Such a setting could also be used to understand the competition between platforms of the sharing economy, for instance, between profit-based and not-for-profit in line with the existing work between proprietary and open source approach in software programming.

Along those lines, a limitation of the existing model is the simple and homogeneous representation of the population. This choice was motivated by our focus on the interplay between monetary and non-monetary motivations, as well as the efficiency of user reviews. Consequently, this model could be used to study some other aspects of the platform governance, particularly when the participants’ characteristics are not uniformly distributed or when the platform has to deal with seasonal activity.

## Figures and Tables

**Fig. 5:**
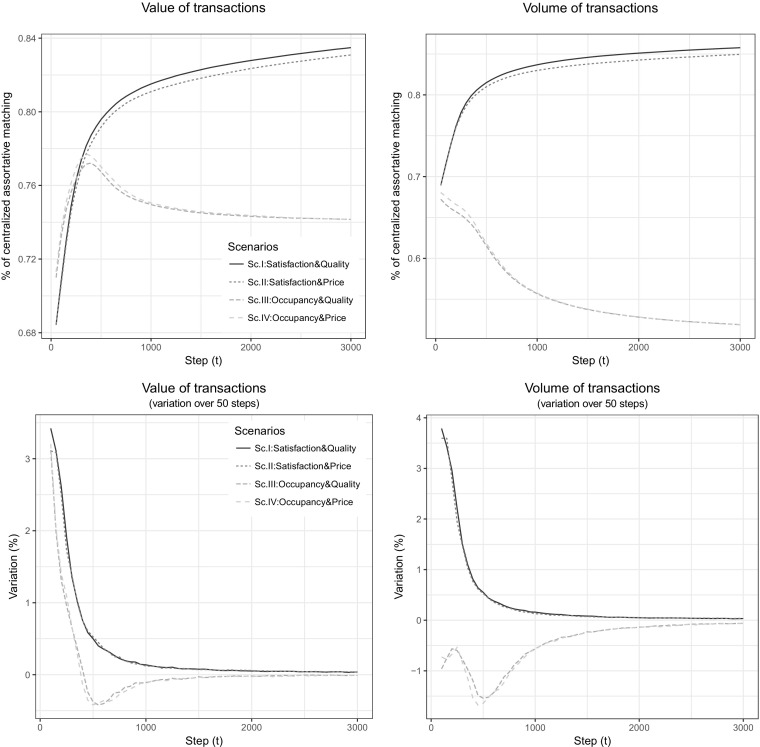
Effect of the number of time steps

**Fig. 6:**
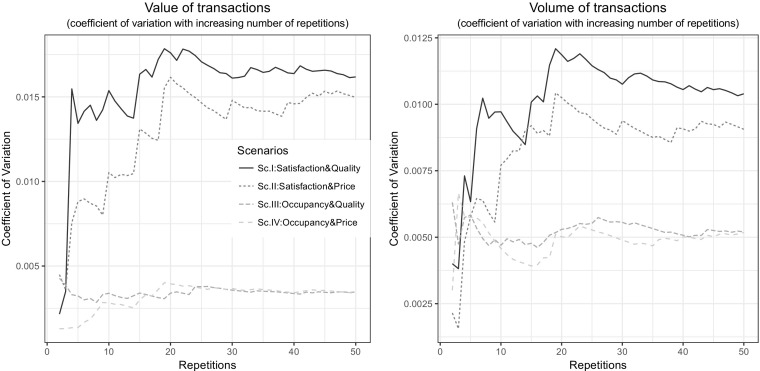
Number of repetitions and coefficient of variation

**Fig. 7:**
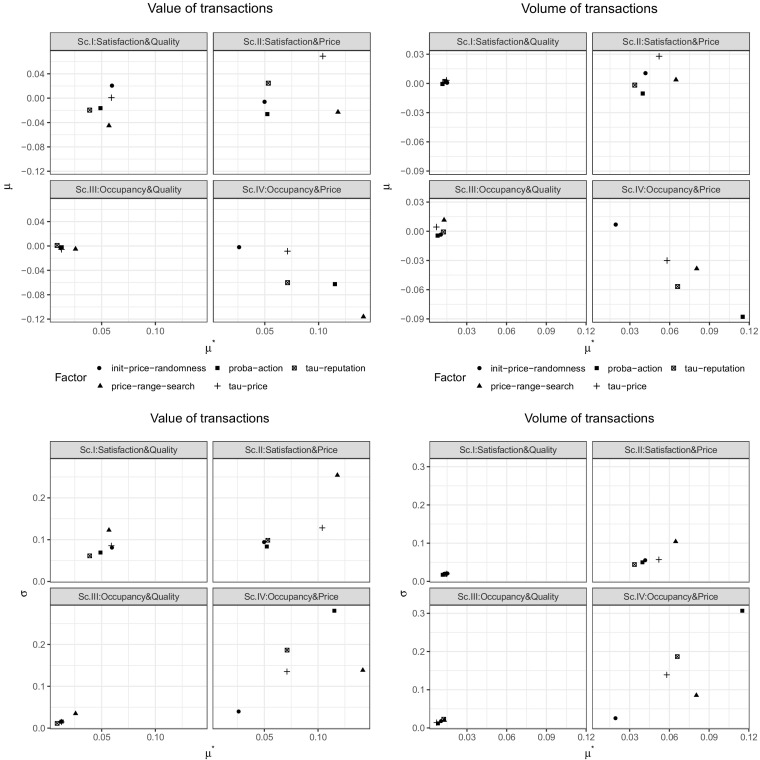
Morris screening method

## Appendix

In the following, we explain the choice of parameter values. Table 3 gives an overview of the variables of interest available in the model.

**Table 3:** Summary of variables, factors and ranges used for the simulations and sensitivity analysis

Name	Type	Range
Value of transactions	Independent	[0;1], i.e. percentage of centralized assortative matching
Volume of-transactions	Independent	[0;1], i.e. percentage of centralized assortative matching
Population *P*	Control	200
Time steps *t*	Control	2000
Repetitions	Control	40
Scenario	Dependent (complex factor)	I (Satisfaction & Quality), II (Satisfaction & Price), III (Occupancy & Quality), IV (Occupancy & Price)
Randomness ratio *γ*	Dependent	Base = 0.5, FF = OAT = [0;1] (5)
Frequency of action *ε*	Dependent	Base = 0.2, FF = [0.18;0.22], OAT = [0.1;0.3] (5)
Price range *r*	Dependent	Base = 2, FF = [1;15], OAT = [0;50] (10)
Tau price	Dependent	Base = 0.1, FF = [0.05;0.25], OAT = [0.00;0.25] (5)
Tau reputation	Dependent	Base = 0.1, FF = [0.05;0.25], OAT = [0.00;0.25] (5)

*Base*, Base value; *FF*, range of full factorial design; *OAT*, range for Morris’ Elementary effect (number of levels between brackets)

i. Number of time steps and number of repetitions

Using the base values of the parameters, we study the evolution of the independent variables over a long period of 3000 time steps. Its evolution is shown in Fig. 5 by reporting the variables themselves (top panel) and the evolution of the variables during periods of 50 steps. When t = 2000, we consider that the variation of the results is almost null. As a trade-off between computational costs and quality of data, we limit the simulation to 2000 time steps.

Following Lorscheid et al. (2012), we control for the stochasticity of the results by using a number of repetitions that stabilizes the coefficient of variation of the independent variables. This stabilization can be observed on Fig. 6 around 40 repetitions.

ii. Local sensitivity analysis: the relative importance of variables

Following Thiele et al. (2014), we perform a local sensitivity analysis by looking at the effect of the dependent factors (or variables) on the independent variables, one factor at a time. Due to the number of inputs and the high-computational cost of the model, we follow the Morris method, which offers the best trade-off between information quality and computational costs. The results are reported on Fig. 7, and the parameter space is created from the full factorial design described in Table 3.

The top panel of Fig. 7 gives an overview of the effect of the factors on the independent variables. This is achieved by the comparison of the two measures *μ* (average effect of each factor using values sampled from the factorial design) and *μ* ^∗^ (similar to *μ*, using the absolute value of the effect). *μ* ^∗^ shows the influence from the factors on the independent variable, while the comparison between *μ* ^∗^ and *μ* tells us if the influence is monotonic. First, the importance of each factor varies with the scenarios, making it difficult to pin down a single factor of interest. For instance, the degree of randomness of the initial price seems to have a strong influence of Scenario IV, but this factor is almost non-influential in other scenarios. Second, the difference of scale between *μ* ^∗^ and *μ* lets us suspect the existence of non-monotonic relationships. For instance, *τ*_*price*_ shows a strong influence on the value of transactions in Scenario I, as measured by *μ* ^∗^. Since its value on the *μ* axis is almost null, we suspect a nonlinear relationship.

On the bottom panel, we observe the interaction between factors measured by *σ* (the standard deviation of the effect *μ*). Again, the sensitivity varies between scenarios. In addition, many factors exhibit a relatively high *σ*, showing the existence of interactions between factors.

iii. Global sensitivity analysis: main effect and interaction effect

Using the effect analysis method proposed by Lorscheid et al. (2012) and implemented by Thiele et al. (2014), we quantify the effect of the different variables, in interaction. For the following work, we use the extremes of the full factorial design shown in Table 3.

The first step of the effect analysis consists in a factorial ANOVA. The Table 4 reports the levels of significance, looking at the main effects by individual factors and the interaction effects of pairs of factor on the two independent variables for the four scenarios. As expected, the variables with limited main effects such as *ε* and *τ*_*reputation*_ have limited interaction effects when coupled with other variables. The second step consists in measuring the strength of the effects (main and interaction). Table 5 reports the effects on the value of transactions and Table 6 reports the effects on the volume. The diagonal (in bold) shows the main effects.

**Table 4:** Levels of significance of main effects and interaction effects measured by factorial ANOVA

	I Satisfaction & Quality		II Satisfaction & Price		III Occupancy & Quality		IV Occupancy & Price	
Factors	value	volume	value	volume	value	volume	value	volume
γ (initial randomness)	***	***	***	***	***	***	***	***
ε (action frequency)	***	***		***	***	***	*	***
r (price range)	***	***	***	***	***	***	***	***
τ_price_	***	***	***	***	***		***	***
τ_reputation_	***	***	***	***	***		***	
γ: ε	**	***	***	***			**	
γ: r	***	***	***	***	***	**	***	***
ε: r					*		**	
γ: τ_price_	***		***	***	***	***	***	*
ε: τ_price_	*	*						
r: τ_price_	***	***	***	***	***		***	***
γ: τ_reputation_	***	***	***	***		*		**
ε: τ_reputation_	*		***	**				
r: τ_reputation_	***	***	***	***	***		***	**
τ_price_: τ_reputation_	***	***		**				

*for p-value <0.05

**for *p*-value <0.01

***for p-value <0.001

**Table 5:** Effect matrix for value of transactions

Factors	γ	ε	r	τ_price_	τ_rep_
Scenario I Satisfaction & Quality					
γ	**−0.0200**	0.0024	0.0185	0.0032	−0.0097
ε		**0.0026**	−0.0011	−0.0019	0.0016
r			**0.0732**	−0.0239	−0.0160
τ_price_				**−0.0253**	−0.0109
τ_rep_					**0.0273**
Scenario II Satisfaction & Price					
γ	**−0.0177**	0.0037	0.0211	0.0035	−0.0099
ε		**0.0010**	−0.0013	−0.0002	0.0029
r			**−0.0316**	0.0327	−0.0238
τ_price_				**0.0325**	−0.0014
τ_rep_					**0.0210**
Scenario III Occupancy & Quality					
Factors	γ	ε	r	τ_price_	τ_rep_
γ	**−0.0057**	0.0003	−0.0031	−0.0047	0.0002
ε		**−0.0013**	−0.0006	−0.0001	0.0001
r			**0.0011**	−0.0060	0.0024
τ_price_				**−0.0052**	−0.0002
τ_rep_					**−0.0062**
Scenario IV Occupancy & Price					
γ	**−0.0067**	0.0007	−0.0030	−0.0038	−0.0002
ε		**−0.0006**	−0.0008	−0.0002	0.0005
r			**−0.0490**	0.0429	0.0021
τ_price_				**0.0440**	−0.0002
τ_rep_					**−0.0064**

Main effects in bold font, interaction effects elsewhere

**Table 6:** Effect Matrix for Volume of transactions

Factors	γ	ε	r	τ_price_	τ_rep_
Scenario I Satisfaction & Quality					
γ	**- 0.0297**	0.0026	0.0087	0.0011	−0.0061
ε		**0.0026**	−0.0005	−0.0011	0.0006
r			**0.1012**	−0.0102	−0.0153
τ_price_				**−0.0113**	−0.0061
τ_rep_					**0.0191**
Scenario II Satisfaction & Price					
γ	**−0.0323**	0.0037	0.0070	0.0038	−0.0075
ε		**0.0019**	−0.0007	−0.0005	0.0016
r			**0.0459**	0.0088	−0.0195
τ_price_				**0.0102**	−0.0017
τ_rep_					**0.0144**
Scenario III Occupancy & Quality					
Factors	γ	ε	r	τ_price_	τ_rep_
γ	**0.0026**	−0.0001	−0.0009	−0.0014	−0.0007
ε		**−0.0052**	0.0001	−0.0001	0.0001
r			**−0.0031**	−0.0002	−0.0004
τ_price_				**−0.0006**	0.0001
τ_rep_					**0.0004**
Scenario IV Occupancy & Price					
γ	**0.0013**	−0.0002	−0.0015	−0.0006	−0.0008
ε		**−0.0047**	0.0004	−0.0004	0.0002
r			**−0.0084**	0.0056	−0.0008
τ_price_				**0.0058**	−0.0001
τ_rep_					**0.0004**

Main effects in bold font, interaction effects elsewhere

## Footnotes

1
https://www.airbnb.com/about/about-us (last visited on August 7, 2017)

2 Full email available at http://blog.airbnb.com/fighting-discrimination-and-creating-a-world-where-anyone-can-belong-anywhere/ (last visited on August 7, 2017)

3
http://www.fastcoexist.com/3046119/defining-the-sharing-economy-what-is-collaborative-consumption-and-what-isnt (last visited on August 7, 2017)

4 Other parameter values have been tested to guarantee the robustness of our results. The results are collected once the platform has stabilized (large *t*); *ε* does not affect the relative results of the scenarios. The number of repetitions (40) is large enough to stabilize the coefficient of variation of the variables of interest. See the Appendix for a more detailed analysis of these values.

5 The point can be verified in the Appendix. The Tables 5 and 6 show that the price range *r* has the highest main effect, by comparison to the other variables in the model.

6
https://www.airbnb.com/help/article/39/what-factors-determine-how-my-listing-appears-in-search-results (last visited on August 7, 2017)
